# Cone-Beam Computed Tomography Assessment of the Prevalence and Association of Pulp Calcification with Dental and Periodontal Pathology: A Descriptive Study

**DOI:** 10.3390/jcm14041373

**Published:** 2025-02-19

**Authors:** José Luis Sanz, Lucía Callado, Stefana Mantale, Jenifer Nicolás, James Ghilotti, Carmen Llena

**Affiliations:** Department of Stomatology, Faculty of Medicine and Dentistry, Universitat de València, Gascó Oliag 1, 46010 Valencia, Spain; jose.l.sanz@uv.es (J.L.S.); luciacallado@gmail.com (L.C.); stefanamantale@gmail.com (S.M.); jennyfernicolas@outlook.es (J.N.); maria.c.llena@uv.es (C.L.)

**Keywords:** cone-beam computed tomography, pulp stone, prevalence, dental caries, dental restoration, alveolar bone loss, orthodontics

## Abstract

**Background/Objectives**: Pulp stones (PSs) are calcified masses, with rounded or oval shapes, ranging from small particles to masses larger than the chamber and/or canals. There are limited data regarding the prevalence of pulp stones in the Iberian population. Our aim was to determine the prevalence of PSs, using CBCT, in an Iberian population, and its association with gender, age, tooth location (arch and hemiarch), dental group, the presence of caries, restorations, alveolar bone loss, and a history of orthodontic treatment. **Methods**: In total, 300 CBCTs were analyzed, selected from the database of the Dental Clinic of the University of Valencia. A total of 5485 teeth were included. The images were obtained by NewTom equipment and visualized using NNT software 11 by a single calibrated examiner in the axial, sagittal, and coronal planes. The Chi-square test, ANOVA, and *t*-test were used to analyze the study variables for a significance level of *p* < 0.05. **Results**: The prevalence of PSs was 88.3% from the total number of patients assessed and 61.2% from the total number of teeth assessed. No differences were found by gender or age. A significant association was found within tooth groups between arches and hemiarches. The proportion of PSs was 3.7 times higher in teeth with caries, 4.7 times higher in teeth with fillings, and 2.3 times higher in teeth with alveolar bone loss. **Conclusions**: PSs were more prevalent in molars. The presence of caries, fillings, and bone loss increased the chance of presenting PSs. Maxillary teeth had a higher prevalence of PSs than mandibular teeth.

## 1. Introduction

Pulp stones (PS) are calcified masses, with rounded or oval shapes, ranging from small particles to masses larger than the pulp chamber and/or canals [1,2]. Although they may be associated with teeth with different pathologies, they also appear in healthy teeth, unerupted teeth, and in both dentitions [3]. The most frequently affected teeth are the first molars, while the least affected teeth are incisors and canines [4].

Tissue aging (both vascular and nervous) [2,5] and the presence of fat deposits in the pulp, where calcification would occur, have been proposed as pathogenic factors for PS development [6]. Caries and restorations behave as irritant factors [7]. Bruxism, which is more prevalent in women, is another irritant factor that could be the reason why some authors find a higher prevalence of PSs among women [6,8]. Other authors do not confirm this association [9]. Orthodontic treatments have also been mentioned as possible irritant factors favoring the appearance of SP [10]. Systemic diseases, such as dentinogenesis imperfecta, dentin dysplasia, Marfan syndrome, William syndrome, or SMAD6 gene polymorphism, have been found to be associated with a higher prevalence of PSs [11]. A high prevalence of PSs has been observed in patients with cardiovascular disease, kidney stones, gallstones, and salivary gland stones. It is hypothesized that the relationship between PSs and other pathological calcifications is due to “calcified nanoparticles”, which could produce a carbonated apatite that would give rise to a biofilm on which mineral aggregates very similar to those found in other human biofilms can be deposited [12].

The prevalence of PSs ranges from 8% to 90%. This large variability is due to the differences in terms of the study population, study type or methodology, and/or sample selection [13]. The most used radiographic method for the detection of PSs is conventional, panoramic, or intraoral radiography [6,7,8]. In recent studies, cone-beam computed tomography (CBCT) is used to provide a three-dimensional image, which can overcome anatomical overlaps. It is, therefore, considered to be a more sensitive tool for the detection of PSs [14,15]. Studies using CBCT have shown higher prevalences versus conventional radiographic modalities, attributing this to the fact that two-dimensional radiographs may underestimate PSs that are less than 200 µm in diameter [6,16].

The aim of this study is to determine the prevalence of PSs, using CBCT, in an Iberian population, and its association with gender, age, tooth location (arch and hemiarch), tooth group, the presence of caries, restorations, bone loss, and a history of orthodontic treatment. The scarce information regarding the prevalence of PSs in the Iberian population acts as the justification for this study.

The null hypothesis that we intend to confirm or reject is that no association will be found between the prevalence of PSs and the presence of dental or periodontal pathology or having undergone orthodontic treatment.

## 2. Materials and Methods

The present observational, descriptive, retrospective, and cross-sectional study was approved by the ethics committee of the Universitat de València (21 February 2021, Registry 1565697) and conducted between March 2021 and June 2024, following the principles of the Declaration of Helsinki.

CBCTs performed for medical reasons were selected from the database of the Dental Clinic of the University of Valencia (Spain). It is a university clinic where patients from all social levels and backgrounds are treated. For the present study, only patients of Iberian origin have been included. Patients between 18 and 86 years of age with at least 20 teeth present were included. CBCT scans of patients with orthodontic appliances, low-quality images that did not allow for the correct assessment of the variables under study, or images in which the two complete dental arches were not visualized, were excluded. Teeth with root resorption, with previous root canal treatment, teeth that could not be assessed due to their position in the arch, teeth with full crowns, metal restorations, and third molars were also excluded.

Images were obtained using NewTom equipment (GIANO HR, Imola, Italy) with the following parameters: 90 Kv, 10 mA and 13 s exposure, voxel size of 100 µm, with an FOV of 11 × 8 cm. The scans were stored in DICOM format and evaluated using NNT software 11 (GIANO HR, Imola, Italy) on an LG monitor (LG Electronics, Madrid, Spain) with a resolution of 1920 × 1080, adjusting brightness and contrast, without ambient light. Images were evaluated in the sagittal, axial, and coronal axes.

The parameters collected were age, gender, tooth (FDI coding), the presence or absence of PSs (defined as a hyperdense image within the pulp chamber or root canals), the presence of caries lesions or fillings (affecting the outer, middle, or inner third of the dentin), and vertical bone loss in mm (measuring in each tooth in a coronal section the distance from the amelocemental junction to the alveolar crest in the area, where the greatest bone loss was observed). If the distance was greater than 3 mm, the tooth was considered to be affected by vertical bone loss.

The sample size was calculated based on the proportion of teeth with PSs of 60%, for a confidence level of 95% and considering an error of 5%, with a required sample of 256 patients.

Statistical analysis was performed using the SPSS 28.0.1.1 package (IBM SPSS, IBM Corp., Armonk, NY, USA). Descriptive data for teeth and patients are presented. The association between the proportion of PSs between the arch and hemiarch was analyzed, considering the patients who had all the teeth from a dental group in the arch or hemiarch and those who only had some teeth, using the Chi-square test. Comparison between qualitative and quantitative variables was performed using ANOVA when comparing more than two groups using Tukey’s test for two-way comparison, or the *t*-test when comparing two groups. Quantitative variables were analyzed using Spearman’s correlation coefficient. In all cases, a significance level of *p* < 0.05 was established.

## 3. Results

All images were evaluated by three investigators, who performed a previous calibration with an experienced radiologist by viewing 50 CBCTs (interobserver Kappa index 0.87–0.89). To confirm consistency during the study period, the observers re-evaluated 10% of the CBCTs in the last month (interobserver Kappa index 0.89–0.92).

A total of 580 CBCTs were reviewed. Following the inclusion and exclusion criteria, 300 were eligible for analysis. The gender distribution was 126 males (42%) and 174 females (58%). The mean age was 53.28 ± 15.57 years; for males, it was 51.02 ± 15.82 years, and for females 54.92 ± 15–21 (*p* = 0.03). A total of 6112 were present; 627 were excluded, leaving 5485 for analysis, as follows: 1186 (21.6%) were molars, 1471 (26.8%) were premolars, 1072 (19.5%) were canines, and 1756 (32.1%) were incisors.

PSs were found in 275 patients (88.3%) and in 3354 teeth (61.2%). The distribution by gender is described in Table 1. The mean number of calcified teeth per patient was 11.17 ± 6.22, being 11.33 ± 5.80 in males and 11.05 ± 6.53 in females (*p* = 0.14). The mean age of the participants with PSs was 53.16 ± 15.60 and 59.33 ± 13.79 (*p* = 0.67) in those without PSs.

The prevalence of PSs by tooth group and location is shown in Table 1. Molars presented 85% of PSs. The molar with the highest prevalence of PSs was 1.6 (90.61%), followed by 4.7 (83.32%). By arch, the prevalence of PSs in the maxillary arch and in maxillary incisors and molars was significantly higher than in the mandibular arch. No differences were found in the hemiarch.

The group aged 18 to 40 years (65 patients) had an average number of teeth with PSs of 13.15 ±6.63, the group aged 41 to 60 years (110 patients) had an average number of teeth with PSs of 11.4 ± 6.04, and the group aged >60 years (125 patients) had an average number of teeth with PSs of 9.93 ± 6.63. The differences between the group aged 18 to 40 years and >60 years were significant (*p* = 0.02). Using Spearman’s correlation coefficient, an inverse and statistically significant correlation was found between age and the number of PSs (rS = −0.24, *p* < 0.001). The association between the number of teeth present and age also yielded an inverse and significant association (rS = −0.44, *p* < 0.001). The correlation between the number of teeth present and the number of PSs was positive and significant (rS = 0.62, *p* < 0.001).

The association by arches and hemiarches in patients with all teeth and in those with only some teeth was calculated using the Chi-square test. In most cases, it was significant in the proportion of PSs between arches and hemiarches (Table 2).

Using ANOVA and Tukey’s post hoc test, the recoded age and the average number of teeth with PSs per arch and hemiarch for all dental groups were compared. In most comparisons, no significant differences were found in the average number of teeth with PSs (Table 3).

In the analysis by gender, the *t*-test was used to compare the average number of PSs per dental group between arches and hemiarches. Only in patients with all maxillary and mandibular molars did females show a significantly higher average (*p* = 0.04) than males: 6.96 ± 1.69/5.77 ± 2.04.

The prevalence of PSs in teeth with caries was 79% and with fillings 80.5% (Table 1). Table 4 summarizes the data on the association between PSs and caries or fillings and their extent. Both the presence of caries and fillings were associated with a higher average number of teeth with PSs.

A prevalence of PSs of 69.7% was found in teeth with bone loss (Table 1). Patients without bone loss had an average number of teeth with PSs of 10.87 ± 8.23 teeth, and in patients with bone loss, 11.20 ± 6.03 (*p* = 0.002). The bone loss in the group of patients with PSs was 9.47 ± 6.53 mm, and in the patients without PSs, it was 1.83 ± 0.71 mm (*p* = 0.002). In molars, the average bone loss was 13.20 ± 8.32 mm (Table 5). All patients with bone loss had some molars with PSs.

A total of 72 patients had worn orthodontics. Although the average number of teeth with PSs was higher for those who had been orthodontic wearers, this difference was not significant (11.87 ± 6.69/11.09 ± 6.17) (*p* = 0.50).

## 4. Discussion

Imaging tests are a non-invasive method that allows for the in vivo analysis of the hard tissues of the maxillofacial area with many elements. Currently, it is the best available technique to evaluate PSs in a clinical investigation [17,18]. In the present study, CBCT was chosen. Its resolution is between 1.25 and 6.5 pl/mm, lower than that of intraoral radiography, which ranges between 15–20 pl/mm.

In the present study, the prevalence of PSs was 88.3% among patients and 61.2% among teeth. These values are similar per patient and higher per tooth compared to other studies [19,20]. Other authors show lower prevalences, 14.2–24.2% per person and 9.5–0.93% per tooth [4,15,17,21,22]. It should be noted that ethnic differences between studies may account for these differences.

In the present study, no association was found between gender and the presence of PSs in most groups, which coincides with the results from other studies [4,22]. However, some authors found a higher prevalence in women, relating it to the higher prevalence of bruxism in women and to hormonal factors [23,24].

The mean age of the patients with PSs did not differ significantly from those without PSs. However, a significant and inverse correlation was found with age in the prevalence of PSs and in the average number of teeth with PSs per patient, with the group aged 18–40 years showing the highest prevalence of PSs. This is justified by the greater number of teeth present in the younger group, which was tested by the analysis of correlations between the number of teeth present and age, which also showed a significant and negative value. However, when analyzing the prevalence of PSs only in patients with all teeth present, the mean number of teeth with PSs was greater in older people than young people when all maxillary and mandibular premolars or all maxillary premolars were analyzed (Table 3). These results are similar to those found by da Silva et al., 2018 [17].

An analysis was made by dental groups and arches compared to the average number of teeth with PSs and their relationship with age, differentiating the patients who presented all the teeth in both arches and hemiarches to eliminate the effect of the greater number of teeth in the younger patients. This analysis made it possible to determine in greater detail how little influence age had on the results of the present analysis. Some studies present a higher prevalence of PSs in the fifth and sixth decades of life, which could be justified as a reactive response of the dentin–pulp complex to the aggressions that have occurred throughout life [22,25]. However, other studies report that age and the reactive process are not sufficient to explain these results [26]. In addition, the prevalence of PSs in the fifth and sixth decades of life is higher than in the fifth and sixth decades of life.

The molar group was found to be significantly more affected by PSs than the rest of the tooth groups, with 1.6 being the most affected tooth. Most authors, regardless of the radiographic method used, support the predilection for PSs in molars and more specifically in the first molar [4,6,7,15,17,19,21,23,26,27,28,29,30,31]. Ranjitkar et al. attributed this to the fact that molars, being the largest in the arch, may have a better blood supply to the pulp, favoring calcification-forming factors [7].

Maxillary teeth were found to be significantly more affected by PSs, with molars and maxillary incisors having significantly more PSs in the maxillary arch than in the mandibular arch. These results are in accordance with previous studies on conventional radiographs and CBCTs [4,6,7,8,20,21,27,28,29,30]. Other authors found no differences between arches [9,17,19,31]. All the reported studies coincide in the bilaterality of calcifications by hemiarches. This coincides with our study, where both a similar prevalence between hemiarches and a significant association in the average number of calcified teeth in both hemiarches were observed, considering both the patients who had all the teeth and those who only had some teeth in arches and hemiarches [4,17,19].

In this study, the prevalence of PSs was 3.7 times higher in patients with caries and 4.7 times higher in teeth with fillings. The average number of teeth with PSs was higher in teeth with caries or fillings, regardless of their extent. In the study by Sezgin et al., the highest prevalence of PSs was found in the medium-depth restorations [21]. Other authors agree that, as the depth of the restoration increased, the likelihood of finding PSs increased [4,17]. Beres et al. propose that PSs have calcium and phosphorus ratios similar to hydroxyapatite and abnormally high concentrations of Zn and Cu similar to carious dentin. Pulp cells probably produce this unstructured mineralization containing high levels of Zn and Cu due to the activity of an antioxidant enzyme (Cu/Zn superoxide dismutase), and therefore, such an environment of oxidative and inflammatory stress would lead to the formation of PSs [32]. Studies by Ranjitkar et al. and Ravanshad et al. found no association between PSs and dental status [7,29].

Periodontal disease is considered to be an irritant factor on the dentin–pulp complex and an inducer of PSs [15]. In our study, the prevalence of PS in teeth with bone loss was 2.3 times higher than in those without bone loss. A significant relationship was established in the average PSs in patients with vertical bone loss greater than 3 mm. A study that selected patients with periodontal disease, without other irritating factors, such as caries or fillings, established a significant prevalence of PSs in patients with periodontal disease, with the risk of developing PSs increasing as the degree of periodontal involvement increased [15]. The pulp tissue and the periodontium are closely related in terms of anatomy and vascularization, and it is proposed that the exposed lateral and accessory canals would allow for the passage of inflammatory agents to the pulp, and due to the oxidative stress produced by the inflammatory environment, PSs would be produced [27]. In the present study, considering the aforementioned results regarding the prevalence of PSs in teeth with dental or periodontal pathology, the null hypothesis was rejected.

Parallelly, no association was found between having worn orthodontics and the prevalence of PSs, in accordance with those found by Kaabi et al. [23]. Other authors have found that the prevalence increased after treatment with fixed orthodontics [33,34]. Although the null hypothesis regarding the absence of an association between having undergone orthodontic treatment and the presence of PSs is accepted, the small number of patients with orthodontics in this study could justify the differences with other authors.

From a clinical point of view, the presence of PSs constitutes a difficulty for root canal treatment. The greater prevalence of PSs observed in maxillary molars can hinder the clear visualization of the pulp chamber radiographically due to their overlapping with anatomical structures. In addition, access to the canals may be made difficult by the presence of PSs, requiring their careful removal before root canal treatment.

It would be interesting to propose new studies in which the association between systemic diseases with calcifications, such as arteriosclerosis or renal calcifications, and the presence of PSs could be evaluated. In addition, longitudinal studies in which the predictive ability of PSs to have other systemic calcifications in the future is determined could be relevant, given that a high prevalence has been found, even in young people.

One limitation of this study is the partial absence of teeth in some patients, due to the difficulty in obtaining the CBCT of patients of different ages with complete dentition. In order to try to minimize the confounding effect that could result from this circumstance, analyses have been made considering, on the one hand, the patients who had all their teeth and those who did not. Parallelly, the impossibility of assessing the presence of PSs in teeth with full crowns or root canal treatment acts as a limitation of the present study. Lastly, the study was carried out on the basis of CBCT performed in a single center; however, it is a university clinic where patients from all social levels and backgrounds attend. CBCTs have been selected from the Iberian population. Larger multicenter studies with representation of people from different ethnicities could provide different information, both on the prevalence of PSs and its association with different pathologic circumstances.

## 5. Conclusions

After analyzing the results, it can be concluded that molars were the teeth with the highest prevalence of PSs, with the right maxillary first molar being the most affected tooth. The prevalence of PSs was higher in the maxillary arch than in the mandibular arch, and in molars and incisors. Age and gender did not influence the prevalence of PSs or the average number of affected teeth in most analyses. The average number of teeth with PSs was similar in the analysis between arcades and hemiarches. The presence of caries, fillings, and bone loss was found to be associated with a higher presence of PSs. The high prevalence of PSs in molars, especially in the maxillae, should alert the clinician of a possible difficulty in the radiographical assessment of the anatomical structures from the dentin–pulp complex and the increased difficulty for root canal access during endodontic practice.

## Figures and Tables

**Table 1:** Prevalence of PSs by gender, dental group, arch, and hemiarch.

	*n*	No PS		PS		*p*
		N	%	N	%	
Total	300	6	2%	294	98%	
Males	126	3	2.4%	123	97.6%	0.49
Females	174	3	1.7%	171	98.3%	
Teeth	5485	2131	38.8%	3354	61.2%	
Incisors	1756	840	47.8%	916	52.2%	<0.05
Canines	1072	440	41%	632	59%	
Premolars	1471	683	46.4%	788	53.6%	
Molars	1186	168	14.2%	1018	85.8%	
Maxillary arch	2628	977	37.17%	1651	62.82%	
Right side	1552	580	37.37%	972	62.62%	0.42
Left side	1076	397	36.89%	679	63.10%	
Mandibular arch	2860	1154	40.34%	1703	59.54%	
Right side	1709	694	40.6%	1012	59.21%	0.35
Left side	1151	460	39.97	691	60.34%	
Maxillary incisors	848	384	45.28%	464	54.71%	0.02
Mandibular incisors	457	228	49.89%	226	49.45%	
Maxillary canines	503	204	40.55%	299	59.44%	0.34
Mandibular canines	569	236	41.47%	333	58.52%	
Maxillary premolars	643	306	47.58%	337	52.41%	0.36
Mandibular premolars	415	187	45.06%	228	54.93%	
Maxillary molars	634	83	13.09%	551	86.9%	0.03
Mandibular molars	268	43	16.04%	225	83.95%	
Teeth with caries	997	209	21%	788	79%	
Coronal third	248	57	23%	191	77%	0.45
Middle third	408	86	21%	322	79%	
Apical third	341	66	19.3%	275	80.7%	
Teeth without caries	4488	1887	42%	2601	58%	
Teeth with restorations	1270	248	19.5%	1022	80.5%	
Coronal third	316	77	24.3%	239	75.7%	0.54
Middle third	606	104	17.1%	502	82.8%	
Apical third	348	67	19.2%	348	80.3%	
Teeth without restorations	4215	1867	44.3%	2348	55.7%	
Teeth with bone loss	2801	848	30.2%	1953	69.7%	0.001
Teeth without bone loss	2684	1281	47.8%	1398	52.2%	

*p*: statistical significance between the variables with PSs.

**Table 2:** Association between arches and hemiarches per patient (Chi-square).

Tooth Groups	*n*	Relation	N	%	*p*
All maxillary and mandibular molars	43	maxillary/mandibular	40	93%	<0.001
All maxillary molars	78	maxi.right/max.left	74	92.3%	0.02
All mandibular molars	70	man.right/man.left	66	92.9%	<0.001
Any maxillary or mandibular molars	263	maxillary/mandibular	203	64.6%	<0.001
Any maxillary molar	235	max.right/max.left	187	60.4%	<0.001
Any mandibular molar	212	man.right/man.left	155	55.2%	0.12
All maxillary and mandibular premolars	69	maxillary/mandibular	52	68.1%	<0.001
All maxillary premolars	96	max.right/max.left	62	53.1%	<0.001
All mandibular premolars	139	man.right/man.left	91	48.9%	<0.001
Any maxillary or mandibular premolars	263	maxillary/mandibular	204	53.65	<0.001
Any maxillary premolar	223	max.right/max.left	112	34.1%	<0.001
Any mandibular premolars	255	man.right/man.left	152	41.6%	<0.001
All maxillary and mandibular canines	213	maxillary/mandibular	153	60.6%	<0.001
All maxillary canines	227	max.right/max.left	140	42.7%	<0.001
All mandibular canines	275	man.right/man.left	151	43.3%	<0.001
Any maxillary or mandibular canines	300	maxillary/mandibular	214	54.7%	<0.001
Any maxillary canine	276	max.right/max.left	178	47.5%	<0.001
Any mandibular canine	294	man.right/man.left	166	43.9%	<0.001
All maxillary and mandibular incisors	155	maxillary/mandibular	121	69%	0.002
All maxillary incisors	177	max.right/max.left	124	58.2%	<0.001
All mandibular incisors	211	man.right/man.left	139	54.5%	<0.001
Any maxillary and mandibular incisors	252	maxillary/mandibular	180	57.1%	<0.001
Any maxillary incisor	235	max.right/max.left	156	52.8%	<0.001
Any mandibular incisor	239	man.right/man.left	152	52.3%	0.12

*p*: statistical significance prevalence of PSs between different positions of the teeth.

**Table 3:** Association between age and the average number of teeth with PSs per patient arch and hemiarch.

**(a) Patients with All Maxillary and Mandibular Molars Present**					**(a) Patients with All Maxillary and Mandibular Premolars Present**					**(a) Patients with All Maxillary and Mandibular Canines Present**					**(a) Patients with All Maxillary and Mandibular Incisors Present**				
	N	Mean ± SD	df/F	*p*		N	Mean ± SD	df/F	*p*		N	Mean ± SD	df/F	*p*		N	Mean ± SD	df/F	*p*
18–40	32	6.28 ± 1.98	2/0.03	0.97	18–40	37	3.22 ± 2.50 ^a^	2/2.97	0.03	18–40	57	2.26 ± 1.39	2/0.92	0.40	18–40	42	4.51 ± 2.15	2/0.33	0.71
41–60	9	6.28 ± 1.89			41–60	21	3.90 ± 2.16			41–60	86	2.12 ± 1.52			41–60	63	4.12 ± 2.47		
>60	2	6 ± 1			>60	11	5.08 ± 1.88 ^a^			>60	70	2.50 ± 1.34			>60	50	4.24 ± 2.29		
Total	43	6.26 ± 1.89			Total	69	3.75 ± 2.37			Total	213	2.28 ± 1.43			Total	155	4.26 ± 2.32		
**(b) Patients with Any Maxillary and Mandibular Molar Present**					**(b) Patients with Any Maxillary and Mandibular Premolar Present**					**(b) Patients with Any Maxillary and Mandibular Canine Present**					**(b) Patients with Any Maxillary and Mandibular Incisor Present**				
18–40	64	4.70 ± 2.68 ^a^	2/15.62	<0.001	18–40	58	3.20 ± 2.34	2/0.47	0.62	18–40	57	2.23 ± 1.35	2/0.41	0.63	18–40	42	4.05 ± 2.40	2/1.41	0.24
41–60	109	3.56 ± 2.16 ^a^			41–60	97	3.01 ± 2.05			41–60	86	2.03 ± 1.43			41–60	63	3.62 ± 2.40		
>60	117	2.80 ± 1.89 ^a^			>60	107	2.86 ± 2.16			>60	70	2.10 ± 1.31			>60	50	3.37 ± 2.36		
Total	290	3.51 ± 2.29			Total	262	3 ± 2.26			Total	213	2.10 ± 1.36			Total	155	3.62 ± 2.42		
**(c) Patients with All Maxillary Molars Present**					**(c) Patients with All Maxillary Premolars Present**					**(c) Patients with All Maxillary Canines Present**					**(c) Patients with All Maxillary Incisors Present**				
18–40	40	3.27 ± 0.93	2/1.29	0.29	18–40	41	1.70 ± 1.40 ^a^	2/3.20	0.04	18–40	57	1.12 ± 0.80	2/2.58	0.07	18–40	41	2.24 ± 1.50	2/1.24	0.29
41–60	23	3.65 ± 0.71			41–60	33	2.06 ± 1.39			41–60	91	1.05 ± 0.82			41–60	73	2.32 ± 1.42		
>60	15	3.46 ± 1.12			>60	23	2.60 ± 1.26 ^a^			>60	79	1.32 ± 0.77			>60	60	1.93 ± 1.51		
Total	78	3.42 ± 0.91			Total	97	2.04 ± 1.39			Total	227	1.16 ± 0.80			Total	174	2.17 ± 1.48		
**(d) Patients with Any Maxillary Molar Present**					**(d) Patients with Any Maxillary Premolar Present**					**(d) Patients with Any Maxillary Canine Present**					**(d) Patients with Any Maxillary Incisor Present**				
18–40	57	2.85 ± 1.09 ^a^	2/12.41	<0.001	18–40	54	1.59 ± 1.39	2/0.15	0.85	18–40	64	1.09 ± 0.77	2/0.44	0.64	18–40	53	2.07 ± 1.49	2/1.91	0.15
41–60	88	2.40 ± 1.05 ^a^			41–60	86	1.45 ± 1.24			41–60	104	1.02 ± 0.78			41–60	89	2.14 ± 1.43		
>60	90	1.95 ± 1.10 ^a^			>60	83	1.51 ± 1.26			>60	108	1.12 ± 0.78			>60	93	1.75 ± 1.36		
Total	235	2.34 ± 1.13			Total	223	1.51 ± 1.26			Total	276	1.08 ± 0.77			Total	235	1.97 ± 1.42		
**(e) Patients with All Mandibular Molars Present**					**(e) Patients with All Mandibular Premolars Present**					**(e) Patients with All Mandibular Canines Present**					**(e) Patients with All Mandibular Incisors Present**				
18–40	46	2.69 ± 1.54	2/2.18	0.11	18–40	56	1.55 ± 1.46	2/0.14	0.86	18–40	64	1.15 ± 0.82	2/0.48	0.61	18–40	49	2.20 ± 1.39	2/1.91	0.14
41–60	32	2.43 ± 1.66			41–60	78	1.64 ± 1.35			41–60	105	1.11 ± 0.84			41–60	83	1.73 ± 1.44		
>60	29	1.89 ± 1.67			>60	80	1.52 ± 1.40			>60	106	1.24 ± 0.77			>60	78	2.10 ± 1.49		
Total	107	2.40 ± 1.63			Total	214	2.00 ± 1.41			Total	275	1.17 ± 0.81			Total	210	1.98 ± 1.46		
**(f) Patients with Any Mandibular Molar Present**					**(f) Patients with Any Mandibular Premolar Present**					**(f) Patients with Any Mandibular Canine Present**					**(f) Patients with Any Mandibular Incisor Present**				
18–40	51	2.70 ± 1.36 ^a,b^	2/6.56	0.002	18–40	58	1.72 ± 1.36	2/0.05	0.95	18–40	65	1.15 ± 0.81	2/0.31	0.73	18–40	54	2.09 ± 1.40	2/1.48	0.23
41–60	85	2.07 ± 1.19 ^a^			41–60	96	1.77 ± 1.30			41–60	108	1.08 ± 0.85			41–60	93	1.68 ± 1.44		
>60	77	1.97 ± 1.03 ^b^			>60	101	1.79 ± 1.23			>60	121	1.16 ± 0.77			>60	93	1.94 ± 1.48		
Total	212	2.18 ± 1.21			Total	255	1.76 ± 1.28			Total	294	1.13 ± 0.81			Total	240	1.88 ± 1.45		

F: F Snedecor. df: degrees of freedom. The same letter in the superscript indicates statistically significant differences in the same column.

**Table 4:** Average number of teeth with PSs, according to the extent of caries and restorations.

Caries				
Caries in the outer third of the dentin	N	Mean ± SD	*p*	d
No	175	9.20 ± 5.91	<0.001	0.86
Yes	125	13.95 ± 5.57		
Caries in the middle third of the dentin				
No	133	9.24 ± 6.56	0.002	0.58
Yes	167	12.72 ± 5.48		
Caries in the inner third of the dentin				
No	167	10.21 ± 6.43	0.03	0.35
Yes	133	12.39 ± 5.75		
Restorations				
Restoration in the external third of the dentin	N	Mean± SD	*p*	d
No	157	9.89 ± 6.68	0.001	0.44
Yes	143	12.59 ± 5.35		
Restoration in the middle third of the dentin				
No	90	8.32 ± 6.84	0.001	0.68
Yes	210	12.40 ± 5.51		
Restoration in the inner third of the dentin				
No	150	9.96 ± 6.44	0.04	0.40
Yes	150	12.39 ± 5.77		

d: Cohen’s d.

**Table 5:** Bone loss in molars (mm).

	**N**	**Mean**	**SD**
MRSM	220	2.63	1.97
MRFM	195	2.30	2.04
MRSM	218	2.61	1.96
MRFM	207	2.68	2.26
MaLSM	198	2.37	2.19
MaLFM	185	1.96	1.83
MaRSM	204	2.34	2.05
MaRFM	195	2.28	2.03
Mean		13.11	8.37

MRSM: maxillary right second molar; MRFM: maxillary right first molar; MLSM: maxillary left second molar; MRSM: maxillary right second molar; MaLSM: mandibular left second molar; MaLFM: mandibular left first molar; MaRSM: mandibular right second molar; MaRFM: mandibular right first molar.

## Data Availability

Data are contained within the article.

## Abbreviations

The following abbreviations are used in this manuscript:

PSs	Pulp stones
CBCT	Cone-beam computed tomography
